# Supplementary material for: Beta-containing bivalent SARS-CoV-2 protein vaccine elicits durable broad neutralization in macaques and protection in hamsters
Source: Commun Med (Lond). 2023 May 26;3:75. doi: 10.1038/s43856-023-00302-z (PMC10212738; doi:10.1038/s43856-023-00302-z)
Supplement: Supplementary file 1 — Supplementary Information [file 43856_2023_302_MOESM1_ESM.pdf]

Beta-containing bivalent SARS-CoV-2 protein vaccine elicits durable broad neutralization in macaques and protection in hamsters

Catherine Berry[1†], Vincent Pavot[1†], Natalie G. Anosova[2], Michael Kishko[2], Lu Li[2], Tim Tibbitts[2], Alice Raillard[1], Sylviane Gautheron[1], Sheila Cummings[3], Dinesh S. Bangari[3], Swagata Kar[4], Caroline Atyeo[5], Yixiang Deng[5], Galit Alter[5], Cindy Gutzeit[6], Marguerite Koutsoukos[7], Roman M. Chicz[2], Valerie Lecouturier[1*]

[1]Sanofi, Marcy l'Etoile, France

[2]Sanofi, Cambridge, MA, USA

[3]Sanofi, Framingham, MA, USA

[4]BIOQUAL Inc, Rockville, MD, USA

[5]Ragon Institute of MGH, MIT, and Harvard, Cambridge, MA, USA

[6]GSK, Rixensart, Belgium

[7]GSK, Wavre, Belgium

[†] These authors contributed equally: Catherine Berry and Vincent Pavot

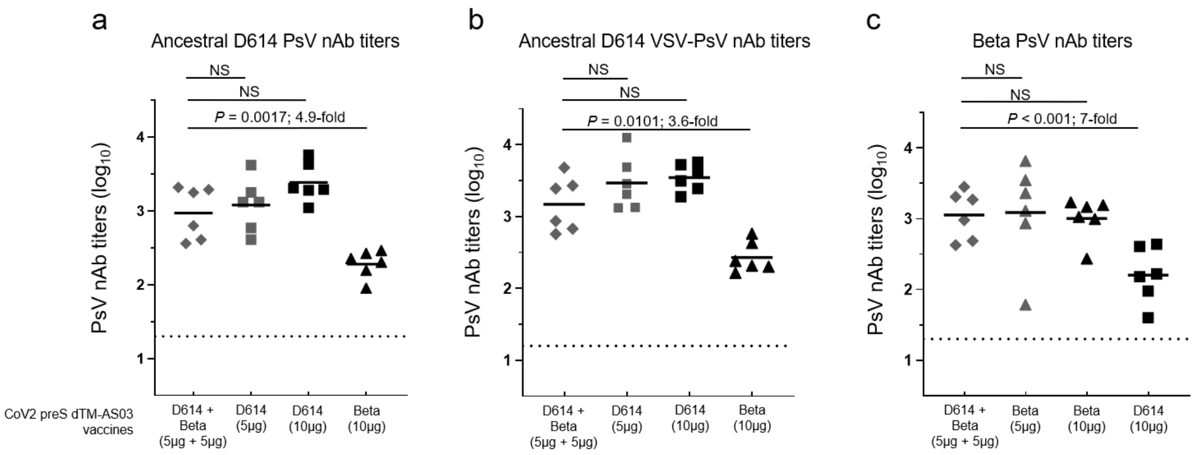

Supplementary Fig. 1 Bivalent CoV2 preS dTM-AS03 (D614 + Beta) induces robust and balanced neutralizing antibody responses against the ancestral SARS-CoV-2 and Beta variant, with no immune interferences, in naïve NHPs. Groups of 6 naïve macaques were immunized twice at 3 weeks interval with the bivalent vaccine (5 µg + 5 µg), monovalent D614 or monovalent Beta (5 µg or 10 µg). Neutralizing antibody titers were measured 2 weeks post second dose. (**a**) ancestral D614 lentivirus pseudovirus nAb titers. (**b**) Ancestral D614 VSV pseudovirus nAb titers. (**c**) Beta lentivirus pseudovirus nAb titers. Individual macaque data are shown (N = 6/group). Bars indicate geometric mean titers (GMT) and horizontal dotted lines indicate the limits of quantification of the assay. NS = non-significant.

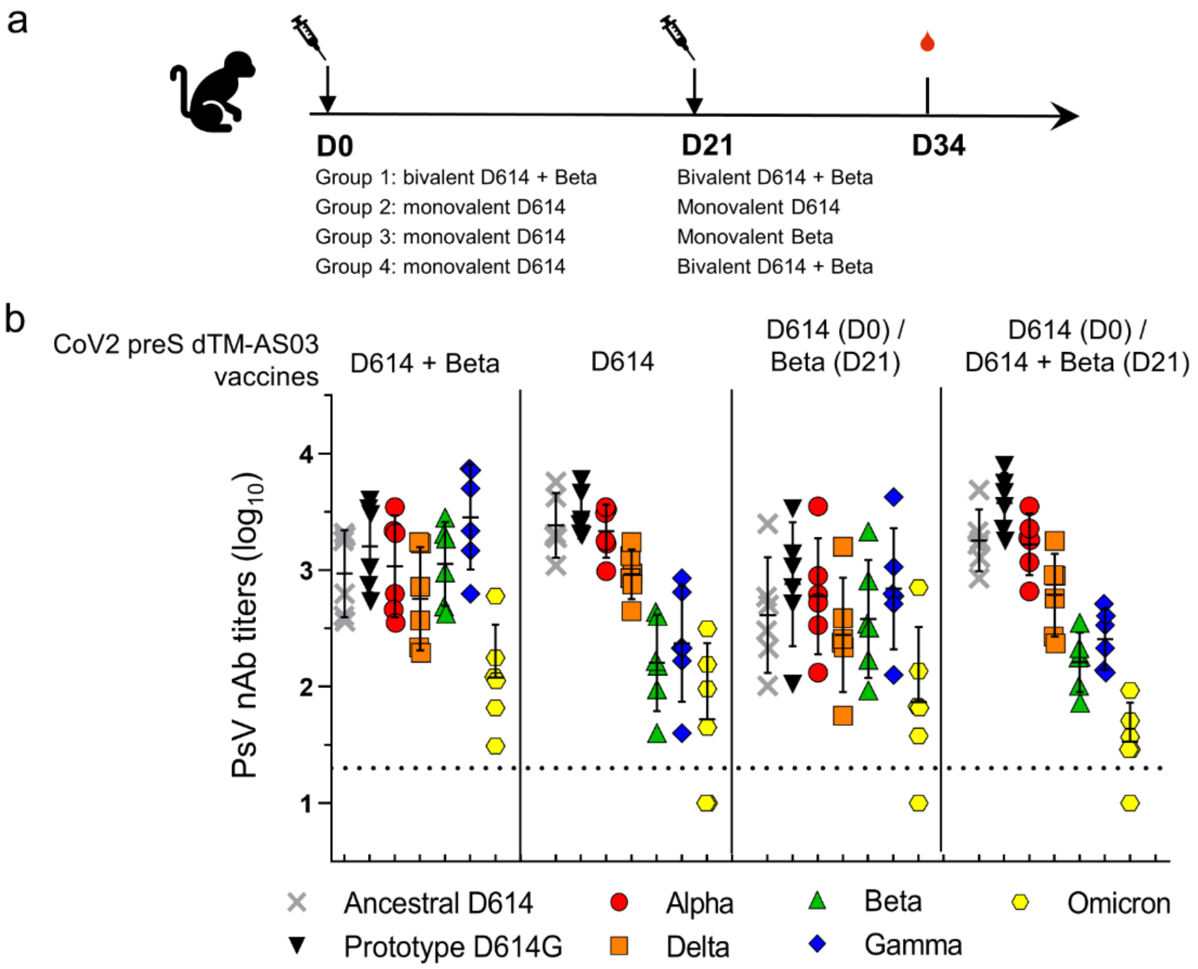

Supplementary Fig. 2 Two dose series with the bivalent CoV2 preS dTM-AS03 (D614 + Beta) induces broader cross-neutralization of variants as compared to heterologous immunization schedules. Groups of six cynomolgus macaques were immunized intramuscularly with CoV2 preS dTM-AS03 vaccine candidates on day 0 (D0) and on day 21 (D21), two groups received either the bivalent D614 + Beta (5 µg + 5 µg) vaccine or the MV D614 (10 µg) vaccine at both injections, two other groups received the MV D614 (5 µg) on D0 and the MV Beta (5 µg) or the BV D614 + Beta (5 µg + 5 µg) on D21 (**a**). Pseudovirus neutralizing antibody titers against SARS-CoV-2 ancestral D614 and prototype D614G, VOC Alpha, Delta, Beta, Gamma and Omicron BA.1 were assayed 2 weeks post second dose (**b**). Individual macaque data are shown (N = 6/group). Means and confidence intervals 95% are indicated. Horizontal dotted line correspond to the inverse of the lowest dilution of the assays.

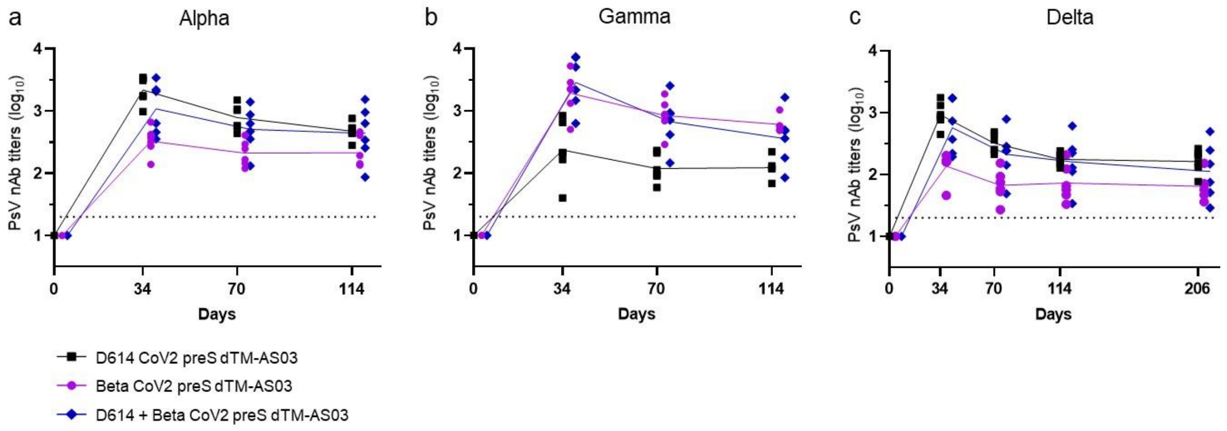

Supplementary Fig. 3 The bivalent CoV2 preS dTM-AS03 (D614 + Beta) vaccine elicits durable nAb titers in NHPs. Pseudovirus neutralizing antibody titers against (**a**) the Alpha variant, (**b**) the Gamma variant and (c) the Delta variant were assessed at different timepoints after immunization in cynomolgus macaques. Individual macaque data are shown (N = 6/group). Connecting lines indicate mean responses and horizontal dotted lines the limits of quantification of the assay.

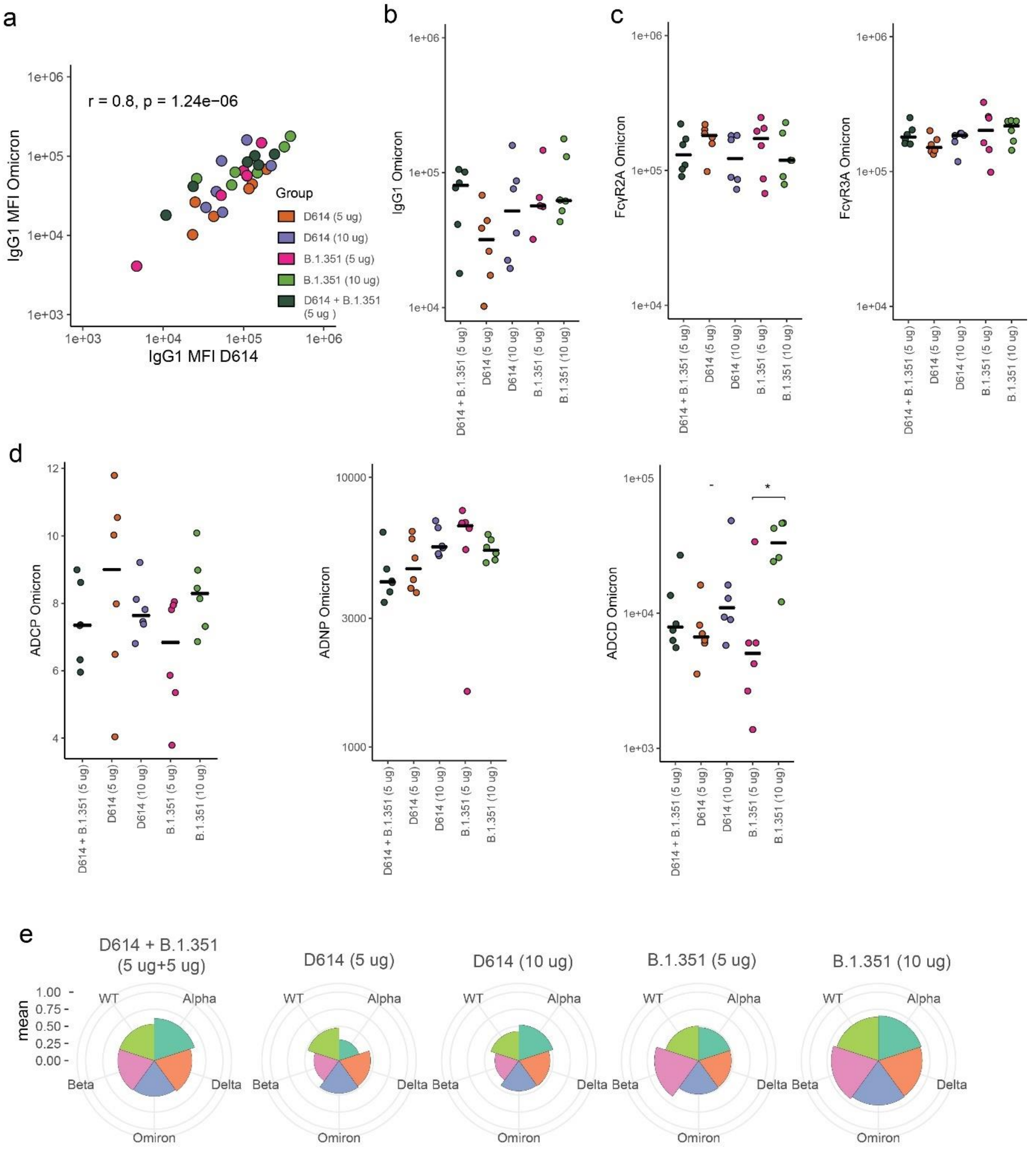

Supplementary Fig. 4 CoV2 preS dTM-AS03 Bivalent (D614 + Beta/B.1.351) vaccine induces Omicron BA.1 IgG and Fc functions. (**a**) The scatter plot shows the IgG1 titer against Omicron BA.1 Spike and the IgG1 titer against D614 spike for each sample. The R and p-value were determined by a Spearman two-tailed test. (**b**) The dot plots show the IgG1 titer against Omicron BA.1 Spike. Significance was determined by a Kruskal-Wallis test. No comparisons were significant. (**c**) The dot plots show the FcR2a-and FcR3a-binding titer against

Omicron BA.1 Spike. Significance was determined by a Kruskal-Wallis test. No comparisons were significant. (**d**) The dot plots show the antibody-dependent cellular phagocytosis (ADCP), antibody-dependent neutrophil phagocytosis (ADNP) and antibody-dependent complement deposition (ADCD) activity against Omicron BA.1 Spike. Significance was determined by a Kruskal-Wallis test, * $P < 0.05$. (**e**) The polar plots show the median percentile rank for each IgG1 Spike measured for the bivalent and monovalent formulations. N = 6/group.

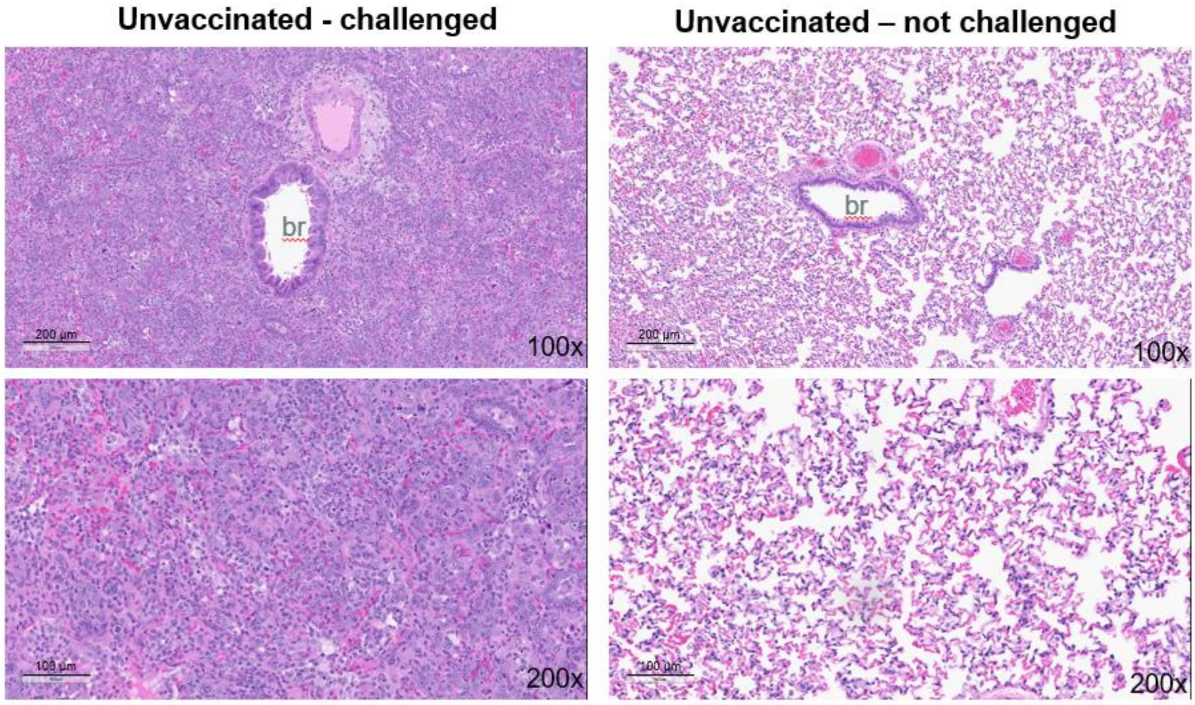

Supplementary Fig. 5 Representative photomicrographs (H&E). Lungs from hamsters receiving buffer only and a viral challenge (left) or unvaccinated and uninfected naïve controls (right). Normal lung architecture is obscured by inflammatory infiltrate, marked type II pneumocyte hyperplasia, cellular debris, and hemorrhage. Naïve lungs exhibit normal pulmonary architecture. Bronchiolar epithelium is hyperplastic (br).
