# Supplementary material for: Beta-containing bivalent SARS-CoV-2 protein vaccine elicits durable broad neutralization in macaques and protection in hamsters
Source: Commun Med (Lond). 2023 May 26;3:75. doi: 10.1038/s43856-023-00302-z (PMC10212738; doi:10.1038/s43856-023-00302-z)
Supplement: Supplementary file 3 — Description of Additional Supplementary Files [file 43856_2023_302_MOESM3_ESM.pdf]

Description of Additional Supplementary File

File Name: Supplementary Data 1

Description: The source data generated in this study
