# Supplementary material for: Beta-containing bivalent SARS-CoV-2 protein vaccine elicits durable broad neutralization in macaques and protection in hamsters
Source: Commun Med (Lond). 2023 May 26;3:75. doi: 10.1038/s43856-023-00302-z (PMC10212738; doi:10.1038/s43856-023-00302-z)
Supplement: Supplementary file 5 — Reporting Summary [file 43856_2023_302_MOESM5_ESM.pdf]

nature portfolio

Corresponding author(s): Valérie Lecouturier

Last updated by author(s): Feb 15, 2023

Reporting Summary

Nature Portfolio wishes to improve the reproducibility of the work that we publish. This form provides structure for consistency and transparency in reporting. For further information on Nature Portfolio policies, see our Editorial Policies and the Editorial Policy Checklist.

Statistics

For all statistical analyses, confirm that the following items are present in the figure legend, table legend, main text, or Methods section.

n/a	Confirmed	
☐	☒	The exact sample size (n) for each experimental group/condition, given as a discrete number and unit of measurement
☐	☒	A statement on whether measurements were taken from distinct samples or whether the same sample was measured repeatedly
☐	☒	The statistical test(s) used AND whether they are one- or two-sided<br>*Only common tests should be described solely by name; describe more complex techniques in the Methods section.*
☒	☐	A description of all covariates tested
☐	☒	A description of any assumptions or corrections, such as tests of normality and adjustment for multiple comparisons
☐	☒	A full description of the statistical parameters including central tendency (e.g. means) or other basic estimates (e.g. regression coefficient) AND variation (e.g. standard deviation) or associated estimates of uncertainty (e.g. confidence intervals)
☐	☒	For null hypothesis testing, the test statistic (e.g. F, t, r) with confidence intervals, effect sizes, degrees of freedom and P value noted<br>*Give P values as exact values whenever suitable.*
☒	☐	For Bayesian analysis, information on the choice of priors and Markov chain Monte Carlo settings
☒	☐	For hierarchical and complex designs, identification of the appropriate level for tests and full reporting of outcomes
☒	☐	Estimates of effect sizes (e.g. Cohen's d, Pearson's r), indicating how they were calculated

Our web collection on statistics for biologists contains articles on many of the points above.

Software and code

Policy information about availability of computer code

Data collection	The data collection and titer calculations were performed using a proprietary software, Sanofi Universal Exporter 2.1. The application takes raw data file from the instruments and transfer them automatically to predifined protocols on the Softmax Pro 6.5.1 GxP software where the calculations are performed, and analyzed according to pre-defined validation criteria.
Data analysis	The analyses were performed on SEG SAS v9.4

For manuscripts utilizing custom algorithms or software that are central to the research but not yet described in published literature, software must be made available to editors and reviewers. We strongly encourage code deposition in a community repository (e.g. GitHub). See the Nature Portfolio guidelines for submitting code & software for further information.

Data

Policy information about availability of data

All manuscripts must include a data availability statement. This statement should provide the following information, where applicable:

- Accession codes, unique identifiers, or web links for publicly available datasets
- A description of any restrictions on data availability
- For clinical datasets or third party data, please ensure that the statement adheres to our policy

Accession codes, and web links for publicly available datasets are provided in the manuscript. The source data generated in this study are included in this paper and the supplementary information.

Human research participants

Policy information about studies involving human research participants and Sex and Gender in Research.

Reporting on sex and gender	*Use the terms sex (biological attribute) and gender (shaped by social and cultural circumstances) carefully in order to avoid confusing both terms. Indicate if findings apply to only one sex or gender; describe whether sex and gender were considered in study design whether sex and/or gender was determined based on self-reporting or assigned and methods used. Provide in the source data disaggregated sex and gender data where this information has been collected, and consent has been obtained for sharing of individual-level data; provide overall numbers in this Reporting Summary. Please state if this information has not been collected. Report sex- and gender-based analyses where performed, justify reasons for lack of sex- and gender-based analysis.*
Population characteristics	*Describe the covariate-relevant population characteristics of the human research participants (e.g. age, genotypic information, past and current diagnosis and treatment categories). If you filled out the behavioural & social sciences study design questions and have nothing to add here, write "See above."*
Recruitment	*Describe how participants were recruited. Outline any potential self-selection bias or other biases that may be present and how these are likely to impact results.*
Ethics oversight	*Identify the organization(s) that approved the study protocol.*

Note that full information on the approval of the study protocol must also be provided in the manuscript.

Field-specific reporting

Please select the one below that is the best fit for your research. If you are not sure, read the appropriate sections before making your selection.

☒ Life sciences ☐ Behavioural & social sciences ☐ Ecological, evolutionary & environmental sciences

For a reference copy of the document with all sections, see nature.com/documents/nr-reporting-summary-flat.pdf

Life sciences study design

All studies must disclose on these points even when the disclosure is negative.

Sample size	Sample size calculation was done on both NHP and hamsters.
Data exclusions	No data were excluded from the analysis
Replication	All assays used for the analytical measures (ELISA and viral neutralization) included internal controls to ensure the reproductibility of the measurements. Repeat measures were performed on subsets of samples to control the reproductibility.
Randomization	Randomization of animals was performed.
Blinding	No blinding was used as sample blinding is not typically used for non-clinical studies, and would have introduced some logistical complexity at different steps (sample collection, storage and analyses).

Reporting for specific materials, systems and methods

We require information from authors about some types of materials, experimental systems and methods used in many studies. Here, indicate whether each material, system or method listed is relevant to your study. If you are not sure if a list item applies to your research, read the appropriate section before selecting a response.

Materials & experimental systems

n/a	Involved in the study	
☐	☒	Antibodies
☐	☒	Eukaryotic cell lines
☒	☐	Palaeontology and archaeology
☐	☒	Animals and other organisms
☒	☐	Clinical data
☒	☐	Dual use research of concern

Methods

n/a	Involved in the study	
☒	☐	ChIP-seq
☒	☐	Flow cytometry
☒	☐	MRI-based neuroimaging

Antibodies

Antibodies used	The WHO International Standard for anti-SARS-CoV-2 immunoglobulin (human) (NIBSC code: 20/136)<br>Human Ig capture antibody (ELISpot kit CTL, CAT# NC1911372)<br>PacBlue anti-CD66b (BioLegend, clone: UCH71)<br>Anti-guinea pig C3 FITC (MpBio)<br>Anti-CD107a–phycoerythrin (PE)–Cy5 (BD Biosciences, lot # 0149826)<br>anti-CD3 Pacific Blue (BD Biosciences, clone G10F5)<br>anti-CD16 allophycocyanin (APC)-Cy5 (BD Biosciences, clone 3G8)<br>anti-CD56 PE-Cy7 (BD Biosciences, clone B159)<br>anti-MIP-1β PE (BD Biosciences)<br>anti-IFN gamma (BD Biosciences)<br>rabbit polyclonal anti-SARS-CoV-2 nucleocapsid antibody (GTX135357, GeneTex, Inc, USA)
Validation	No validation was performed. Antibodies were all from commercial source.

Eukaryotic cell lines

Policy information about cell lines and Sex and Gender in Research

Cell line source(s)	Vero TMPRSS2 (obtained from Adrian Creanga, Vaccine Research Center-NIAID)<br>293T-hsACE2 clonal cells (Integral Molecular, Cat# C-HA102)<br>THP-1 cells (ATCC)
Authentication	All cell lines were from commercial source
Mycoplasma contamination	The cell lines were not tested for mycoplasma after receipt
Commonly misidentified lines<br>(See ICLAC register)	No commonly misidentified cell lines were used

Animals and other research organisms

Policy information about studies involving animals; ARRIVE guidelines recommended for reporting animal research, and Sex and Gender in Research

Laboratory animals	Cynomolgus macaques aged 2-8 years, males and females<br>Female Golden Syrian hamsters aged of 6-8 weeks
Wild animals	The study did not invlolve wild animals
Reporting on sex	Female Golden Syrian hamsters aged of 6-8 weeks were randomized based on weight.<br>Cynomolgus macaques aged 2-8 years, were randomized based on sex, age and weight. Groups were composed of 6 animals including 2 or 3 females and 4 or 3 males.
Field-collected samples	This study did not involve samples collected from field
Ethics oversight	Animal experiments were conducted in compliance with all pertinent US National Institutes of Health regulations according to approved animal protocols from the Institutional Animal Care and Use Committee (IACUC) at the research facilities. The NHP study was performed at the University of Louisiana at Lafayette New Iberia Research Center and the hamster study was performed at Bioqual.

Note that full information on the approval of the study protocol must also be provided in the manuscript.
